# Galectin-9/TIM-3 as a Key Regulator of Immune Response in Gliomas With Chromosome 1p/19q Codeletion

**DOI:** 10.3389/fimmu.2021.800928

**Published:** 2021-12-08

**Authors:** Guanzhang Li, Ruoyu Huang, Wenhua Fan, Di Wang, Fan Wu, Fan Zeng, Mingchen Yu, You Zhai, Yuanhao Chang, Changqing Pan, Tao Jiang, Wei Yan, Hongjun Wang, Wei Zhang

**Affiliations:** ^1^ Department of Molecular Neuropathology, Beijing Neurosurgical Institute, Capital Medical University, Beijing, China; ^2^ Department of Neurosurgery, Beijing Tiantan Hospital, Capital Medical University, Beijing, China; ^3^ Center of Brain Tumor, Beijing Institute for Brain Disorders, Beijing, China; ^4^ China National Clinical Research Center for Neurological Diseases, Beijing, China; ^5^ Chinese Glioma Genome Atlas Network (CGGA) and Asian Glioma Genome Atlas Network (AGGA), Beijing, China; ^6^ Department of Neurosurgery, The First Affiliated Hospital of Nanjing Medical University, Nanjing, China; ^7^ Department of Neurosurgery, The Second Affiliated Hospital of Harbin Medical University, Harbin, China

**Keywords:** glioma, 1p/19q codeletion, DNA repair functions, immune checkpoint, prognosis prediction

## Abstract

Gliomas with chromosome 1p/19q codeletion were considered a specific tumor entity. This study was designed to reveal the biological function alterations tightly associated with 1p/19q codeletion in gliomas. Clinicopathological and RNA sequencing data from glioma patients were obtained from The Cancer Genome Atlas and Chinese Glioma Genome Atlas databases. Gene set variation analysis was performed to explore the differences in biological functions between glioma subgroups stratified by 1p/19q codeletion status. The abundance of immune cells in each sample was detected using the CIBERSORT analytical tool. Single-cell sequencing data from public databases were analyzed using the t-distributed stochastic neighbor embedding (t-SNE) algorithm, and the findings were verified by *in vitro* and *in vivo* experiments and patient samples.We found that the activation of immune and inflammatory responses was tightly associated with 1p/19q codeletion in gliomas. As the most important transcriptional regulator of Galectin-9 in gliomas, the expression level of CCAAT enhancer-binding protein alpha in samples with 1p/19q codeletion was significantly decreased, which led to the downregulation of the immune checkpoints Galectin-9 and TIM-3. These results were validated in three independent datasets. The t-SNE analysis showed that the loss of chromosome 19q was the main reason for the promotion of the antitumor immune response. IHC protein staining, *in vitro* and *in vivo* experiments verified the results of bioinformatics analysis. In gliomas, 1p/19q codeletion can promote the antitumor immune response by downregulating the expression levels of the immune checkpoint TIM-3 and its ligand Galectin-9.

## Introduction

Gliomas, the most common and lethal primary intracranial tumor in adults, are characterized by strong invasiveness and a high recurrence rate (1–3). Tumor-specific molecular alterations, such as isocitrate dehydrogenase 1 (IDH1) mutation and the codeletion of chromosome arms 1p and 19q (1p/19q codeletion), could serve as prognostic indicators or therapeutic targets for glioma patients (4–6). These biomarkers were also included in the 2016 World Health Organization (WHO) Classification of Tumors of the Central Nervous System (CNS), providing an important theoretical foundation for the diagnosis and individualized treatment of gliomas (7). However, the specific molecular mechanism by which 1p/19q codeletion affects the biological characteristics and clinical prognosis of gliomas remains unclear.

Chromosome 1p/19q codeletion is a representative event in the oligodendroglial histologic type of gliomas and is strongly associated with overall survival of glioma patients (8–10). In general, glioma patients with 1p/19q codeletion tend to have a better prognosis (8, 11). Patients with 1p/19q codeletion are more sensitive to chemotherapy with alkylating agents such as temozolomide (TMZ) (12). The loss of chromosome 1p or 19q inevitably results in significant reductions in the expression of multiple genes located on these regions; thus, 1p/19q codeletion can cause significant abnormalities in the biological function of gliomas (13). The remolding of the tumor immune environment plays an essential role in chemotherapy resistance of various cancers (14). Thus, we hypothesized that 1p/19q codeletion may improve glioma sensitivity to postoperative treatment by regulating the tumor immune microenvironment.

This study comprehensively analyzed the alterations in biological functions associated with 1p/19q codeletion in gliomas by gene set variation analysis (GSVA) (15). We observed differences in immune system process between patients according to 1p/19q codeletion status. To further clarify the specific molecular mechanism of 1p/19q codeletion remodeling of the immune microenvironment of gliomas, CIBERSORT was applied, revealing seven immune metagenes (16, 17). The results suggested that 1p/19q codeletion upregulated immune response by reducing the expression of TIM-3 and its ligand Galectin-9 but had no significant effect on immune cell infiltration. We also found that the transcription factor CCAAT enhancer-binding protein alpha (CEBPA), located on chromosome 19q, played a key role in regulating the expression level of Galectin-9 in both lower-grade glioma (LGG) and glioblastoma (GBM). These results indicate that the loss of chromosome 19q in glioma patients with 1p/19q codeletion could significantly inhibit Galectin-9 expression and the immunosuppressive function of TIM-3. In conclusion, this is the first integrative study to illustrate the association between 1p/19q codeletion and the immune microenvironment of gliomas, which may provide a more accurate assessment of individualized clinical management in patients with gliomas. 

## Methods

### Sample and Databases

This study was approved by Beijing Tiantan Hospital Institutional Review Board (IRB). Written informed consents were obtained from the patients (or their families).

This study included a total of 368 patients with data on transcriptome sequencing and 1p/19q codeletion status. The details of sample acquisition and sequencing were described previously (18). Pathological diagnosis was confirmed by at least two neuropathology experts in the Neuropathology Department of Beijing Neurosurgical Institute. The tumor 1p36 and 19q13 statuses were determined using fluorescent *in situ* hybridization analysis of formalin-fixed, paraffin-embedded blocks. The CGGA project performed transcriptome sequencing on two batches of glioma samples at different periods, named the CGGA database and the CGGA New database respectively. These two databases are mutually independent databases and can be used as an independent verification database for each other. Sequence data from the Cancer Genome Atlas (TCGA) mRNA-seq database were downloaded from public databases (https://cancergenome.nih.gov). The clinical information of the patients is summarized in **Table 1**.

**Table 1:** Clinical information of patients.

Characteristics	No. of Patients (CGGA)		No. of Patients (CGGA New)		No. of Patients (TCGA)	
	Codel	Non-Codel	Codel	Non-Codel	Codel	Non-Codel
***Age at diagnosis***						
Mean	38.5	38.1	41.3	38.9	43.5	37.8
Standard Deviation	7.4	8.2	9.3	8.9	12.3	11.7
***Gender***						
Male	29	27	25	46	44	61
Female	15	19	26	24	38	59
***Histology***						
Astrocytoma	0	31	5	30	2	44
Oligodendroglioma	22	1	16	3	63	31
Oligoastrocytoma	22	14	30	37	17	45
***WHO Grade***						
II	44	46	51	70	82	120
III or IV	0	0	0	0	0	0
***IDH1 Mutation***						
Mutation	44	46	51	70	82	120
Wildtype	0	0	0	0	0	0
***Survival Time***						
Range (Days)	181-4143	19-4163	127-4075	41-4374	2-5466	1-6331
Median (Days)	3197	552.5	2703	664	439	373

### Availability of Data and Material

The sequencing data, clinical data and molecular pathology data of all patients were uploaded to the CGGA portal (http://cgga.org.cn/). All data related to this research are available on reasonable request from the first and corresponding authors.

### GSVA

A total of 7,345 biological functional enrichment scores calculated independently for each patient were generated based on default parameters in the *gsva* package in R, as described previously (19). Subsequently, all negatively regulated BPs have been removed. Based on the remaining 6613 biological processes, the biological functions related to 1p/19q codel status were examined. The gene list for each biological function is available from the AmiGO 2 Web portals (http://amigo.geneontology.org).

### Immune Cells and Cytokine Enrichment Scores

The abundance of immune cells in each patient was estimated from transcriptome sequencing data using CIBERSORT analytical tool developed by Newman et al. (16). The calculation was performed online using default parameters (https://cibersort.stanford.edu). The calculation of cytokines scores and the corresponding gene list were calculated by the GSVA algorithm as described previously (18, 20). Subsequently, the significance of the difference between the enrichment score of two groups was verified by *Student’s t-test*.

### Single-Cell Sequencing Analysis

Single-cell sequencing and molecular pathology data were downloaded from the Gene Expression Omnibus databases (https://www.ncbi.nlm.nih.gov/gds/?term=GSE70630 and https://www.ncbi.nlm.nih.gov/gds/?term=GSE89567). Based on the cell markers from the Cellmarker website (http://biocc.hrbmu.edu.cn/CellMarker), the cells for each patient were grouped independently using the t-distributed stochastic neighbor embedding (t-SNE) algorithm in the R environment.

### Immunohistochemical (IHC) Staining

Paraffin-embedded tissues were obtained from the CGGA sample bank. The IHC experimental procedures and scoring methods were as reported previously (21). The antibodies for IHC staining included those for CEBPA (Abcam, ab140479, 1:200), Galectin-9 (Proteintech, 17938-1-AP, 1:100), and TIM-3 (Proteintech, 60355-1-lg, 1:1000).

### Cell Isolation and Culture

293T and U87 cell lines were obtained from the Institute of Biochemistry and Cell Biology, Chinese Academy of Science. 293T and U87 cell lines were cultured in medium containing DMEM (Gibco) supplemented with 10% FBS (Gibco). T cells were obtained from a healthy adult male donor. Isolation and culture of T cells was performed following the protocol as previously described (22).

### siRNA Transfection

The CEBPA siRNA and the corresponding negative control plasmid were purchased from syngentech (Beijing, China). 293T cells were transfected with siRNA using X-tremeGENE HP DNA Transfection Reagent (Roche, Switzerland) according to instructions exactly. The transfection efficiency was determined by the proportion of fluorescence-labeled cells.

### Western Blotting

The extraction of total cell protein and the Western Blotting experiment were carried out as previous described (23). Antibodies for Western Blotting: CEBPA antibody (Abcam, ab140479, 1:1000), Galectin-9 antibody (proteintech, 17938-1-AP, 1:500), TIM-3 antibody (proteintech, 60355-1-lg, 1:1000), GAPDH antibody (proteintech, 60004-1-lg, 1:5000).

### Flow Cytometry

The surgically removed tumor tissue was washed with phosphate-buffered saline and immediately immersed in DMEM medium and transported to the laboratory within half an hour. Then, the tumor sample was fully mechanical and enzymatic dissociated into single cells. After the dead cells removed by Dead Cell Removal Kit (miltenyi, 130-090-101), the remaining cells were used for flow cytometry. Antibodies for flow cytometry: TIM-3 antibody (1:100, BioLegend, 345012), Galectin-9 antibody (1:100, BioLegend, 348911), CD3 antibody (1:100, STEMCELL, 60011AZ.1).

### *In Vivo* Xenograft Growth

Animal experiments were performed at the animal laboratory of Beijing Neurosurgical Institute according to NIH guidelines. Luciferase labeled glioma tumor cells with/without CEBPA knock down and T cells were mixed (1:1, totally 4 × 10^5^ cells in 5 μL of PBS) and then transplanted into the right hemisphere of NOD-Prkdcs^cid^ Il2rg^null^ mice (23). The growth of intracranial tumors in mice was monitored weekly by *in vivo* fluorescence imaging.

### Statistical Analysis

The statistical analysis and visualization in this study were performed using R (https://www.r-project.org/, v3.5.0), IBM SPSS Statistics for Windows, version 25.0 (IBM Corp., Armonk, NY) and GraphPad Prism version 8.0 (La Jolla, CA). After applying the homogeneity test of variance, significant differences between two groups of normally distributed data were verified by the Student’s t-test. Significant correlations between two groups of normally distributed data were verified using the Pearson’s correlation analysis. For all statistical methods, p<0.05 indicated significant differences.

## Results

### Gliomas With 1p/19q Codeletion Show a Unique Tumor Immune Status

**Figure 1:**
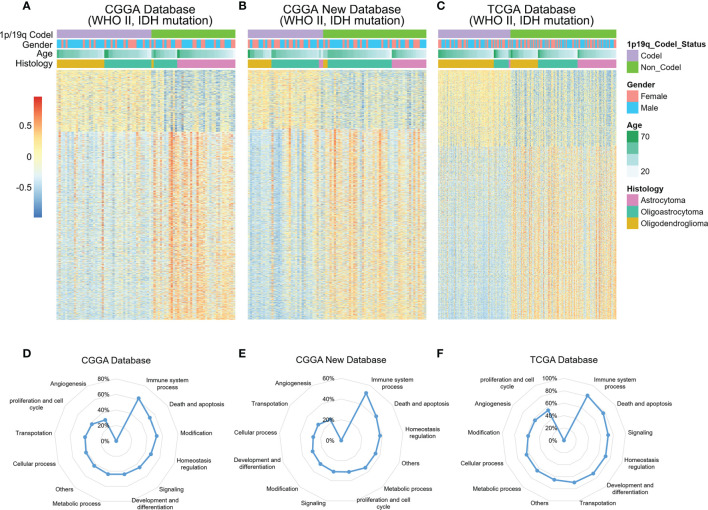
Relationships between chromosome 1p/19q codeletion and altered biological processes in glioma. **(A–C)** Heatmap showing the enrichment patterns of biological processes associated with 1p/19q codeletion status in the CGGA, CGGA New and TCGA databases. **(D–F)** Alterations in different classifications of biological functions in gliomas samples with 1p/19q codeletion.

Chromosomal 1p/19q codeletion, as a genomic variation at the chromosome level, contribute to dysfunction in tumor biological processes (11, 24). To explore the potential functional characteristics of 1p/19q codeleted tumors, the enrichment scores of 6,613 biological functions were obtained through GSVA of the CGGA and TCGA RNA-seq databases. As shown in **Figures 1A**–**C**, more than 3,000 biological functions showed significant differences between 1p/19q codeleted and non-codeleted tumors. After classifying these functions, we found that the immune system process was most tightly correlated with the 1p/19q codeletion status in the glioma samples (**Figures 1D**–**F**). These results indicated that 1p/19q codeletion may play an essential role in the regulation of the tumor immune microenvironment of gliomas.

### Overview of the Immune Process in Tumors With Different 1p/19q Codeletion Statuses

**Figure 2:**
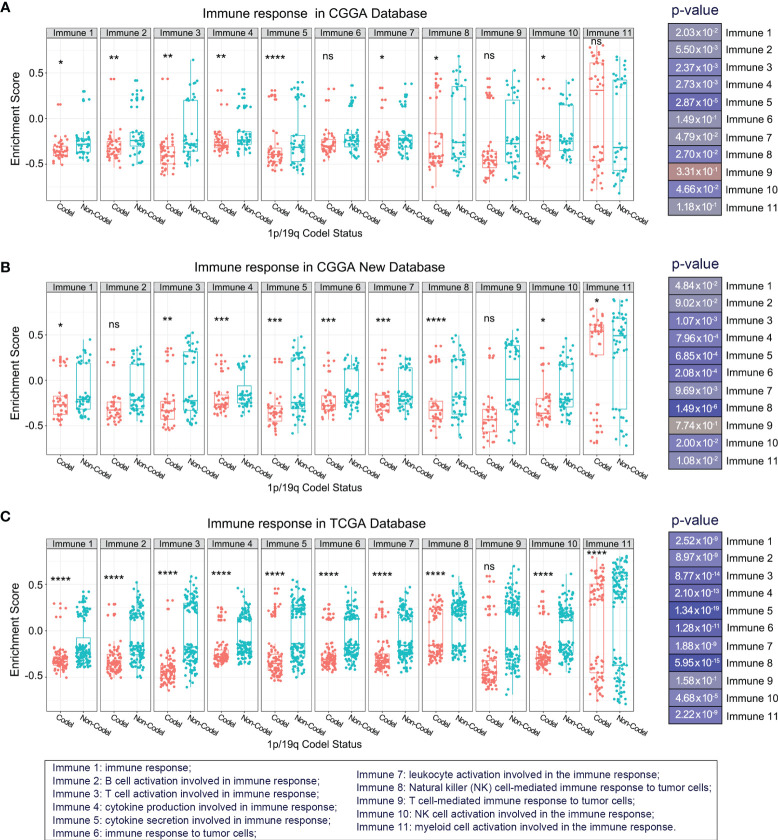
Overview of the differences in kernel immune response in glioma patients stratified by 1p/19q codeletion status in the CGGA **(A)**, CGGA New **(B)**, and TCGA **(C)** databases. The significance of the difference between the two groups was verified by *Student’s t-test*. *^ns^p > 0.05*, ^*^p < 0.05, ^**^p < 0.01, ^***^p < 0.001, ^****^p < 0.0001.

To overview the differences in immune response between 1p/19q codeleted and non-codeleted samples, we included the top 11 critical immune system processes. Student’s t-tests were performed to evaluate the differences in enrichment scores between stratified patients. The results suggested that most tumor-related immune responses were tightly correlated with 1p/19q codeletion status in the CGGA database, especially the activation of T lymphocytes, T cell and natural killer (NK) cell-mediated immune response to tumor cells, as well as the production and secretion of cytokines (**Figure 2A**). These analyses were also performed in the CGGA NEW and TCGA RNA-seq database for validation, with similar results (**Figures 2B, C**). These results indicated that 1p/19q codeletion status had a significant effect on T lymphocytes, NK cells, and cytokine-mediated antitumor immunity.

### 1p/19q Codeletion Status Was Associated With Tumor Inflammatory Activities

**Figure 3:**
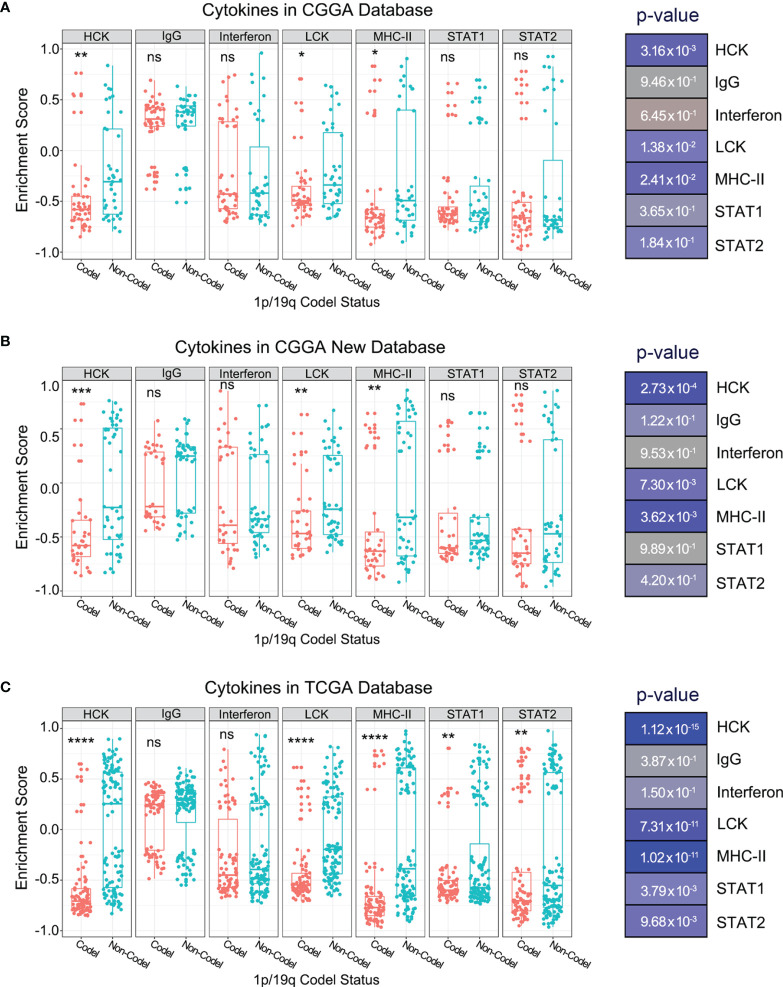
Enrichment scores of seven immune and inflammatory-related metagenes in glioma patients with different 1p/19q codeletion status in the CGGA **(A)**, CGGA New **(B)**, and TCGA **(C)** databases. The significance of the difference between the two groups was verified by *Student’s t-test*. *^ns^p > 0.05*, ^*^p < 0.05, ^**^p < 0.01, ^***^p < 0.001, ^****^p < 0.0001.

Infiltrating immune cells are a vital component of the tumor microenvironment, which has diverse roles in glioma biology (25). Thus, to further explore whether there was a significant difference in immune cell infiltration between 1p/19q codeletion and 1p/19q non-codeletion samples, we evaluated the abundance of various types of immune cells in the CGGA and TCGA databases by CIBERSORT. The results showed significant differences between stratified patients for only a few types of immune cells; however, these differences could not be mutually verified in the three databases (**Supplementary Figures 1A**–**C**). This finding indicates that 1p/19q codeletion status may not regulate the tumor immune microenvironment by affecting the infiltration of immune cells in gliomas. To further investigate the function of 1p/19q codeletion in the tumor immune microenvironment of gliomas, we assessed seven metagenes. As we described previously, these seven metagenes represent several types of inflammation and immune responses (18). The results showed significantly reduced enrichment scores for hematopoietic cell kinase, lymphocyte-specific protein tyrosine kinase, and major histocompatibility complex II (MHC-II) in patients with 1p/19q codeletion (**Figures 3A**–**C**), suggesting the activation of the immunological functions of lymphocyte and antigen-presenting cells.

### Specific Reduction in the Expression of TIM-3 and Its Ligands in Gliomas With 1p/19q Codeletion

**Figure 4:**
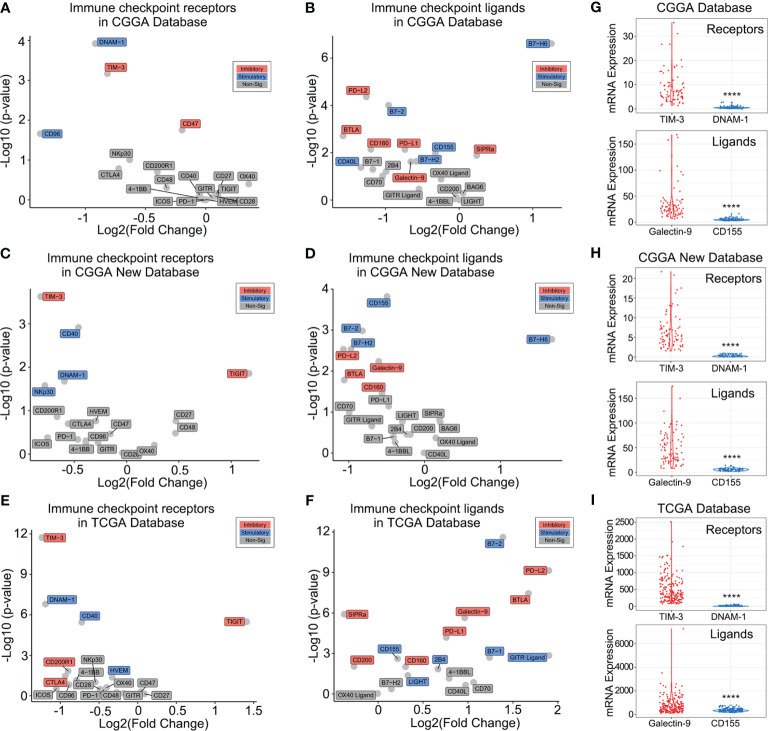
Association between 1p/19q codeletion and immune checkpoints. **(A–F)** Differences in immune checkpoint expression in glioma patients with different 1p/19q codeletion status in the CGGA **(A, B)**, CGGA New **(C, D)**, and TCGA databases **(E, F)** databases. **(G–I)** TIM-3/Galectin-9 and DNAM-1/CD155 expression levels in the CGGA **(G)**, CGGA New **(H)**, and TCGA **(I)**. The significance of the difference between the two groups was verified by *Student’s t-test*. ^****^p < 0.0001.

Our previous studies showed that immune checkpoints such as programmed cell death protein 1 (PD1) and TIM-3 play a crucial role in the regulation of the immune and inflammatory response in glioma (18, 20). This study investigated multiple immune checkpoint receptors and their ligands to further explore the effect of 1p/19q codeletion on the expression level of immune checkpoints. We observed significant differences in some immune checkpoint receptors and their ligands in patients stratified according to 1p/19q codeletion status in the CGGA database (**Figures 4A, B**). For further validation, similar analyses were performed in the CGGA NEW database and TCGA database (**Figures 4C**–**F**). The results showed that only TIM-3 and DNAX accessory molecule-1 (DNAM-1) with their respective ligands showed consistent expression differences between stratified patients across all databases (**Figures 4A**–**F**). However, the expression level of DNAM-1 and its ligand CD155 in glioma was very low, indicating that they may play a limited role in regulating the glioma tumor immune microenvironment (**Figures 4G**–**I**). In contrast, high expression levels of TIM-3 and its ligand Galectin-9 were observed in glioma samples (**Figures 4G**–**I** and **Supplementary Figure 2**). Thus, we hypothesized that 1p/19q codeletion may change the immune microenvironment of glioma by affecting TIM-3 or Galectin-9 expression levels.

### Decreased Expression of TIM-3 and Its Ligands in 1p/19q Codeletion Tumors Owing to Decreased CEBPA

**Figure 5:**
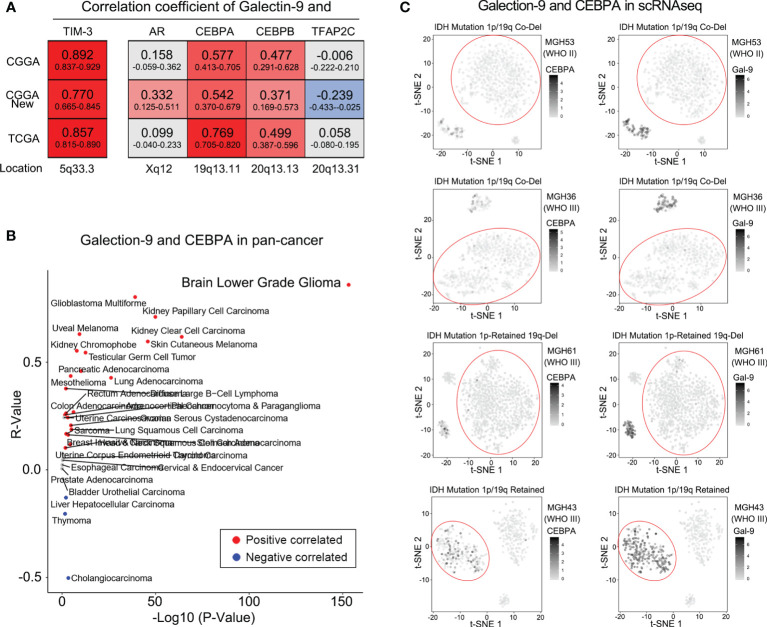
1p/19q codeletion downregulated Galectin-9 expression *via* CEBPA. **(A)** Correlations between Galectin-9 and transcription factors by Pearson correlation analysis in the CGGA and TCGA databases. **(B)** Correlation between Galectin-9 and CEBPA detected by Pearson correlation analysis in the TCGA pan-cancer database. **(C)** t-SNE analysis performed to evaluate the association between Galectin-9 expression and the loss of chromosome 19q.

To explore whether the differential expression of TIM-3 and its ligands in different 1p/19q codeletion status was independent of clinical characteristics, the relationship between them was studied. The results showed that gender, age, total number of tumor involved regions, and the degree of surgical resection are not related to the expression of Galectin-9 and TIM-3 (**Supplementary Figures 3**–**5**). We further investigated the specific molecular mechanism by which 1p/19q codeletion affected TIM-3 and Galectin-9 expression levels. Relevant studies confirmed that four transcription factors (AR, CEBPA, CEBPB, and TFAP2C) are involved in the regulation of Galectin-9 gene expression (26–28). We found that, among these four transcription factors, only CEBPA located on chromosome 19q13.11 was tightly correlated with Galectin-9 expression level in both CGGA and TCGA databases (**Figure 5A** and **Supplementary Figure 6**). Furthermore, we downloaded a pan-cancer database from a public website to further explore whether there are similar phenomena in other kinds of tumor tissues. As shown in **Figure 5B**, compared with other tumor samples, CEBPA and Galectin-9 were most closely correlated in lower-grade glioma (LGG, WHO grades II and III). They were also tightly correlated in glioblastoma multiforme (GBM, WHO grade IV). These results indicated differences in the transcription factors regulating Galectin-9 expression in different tumor tissues. Among them, CEBPA played a key role in the regulation of Galectin-9 expression in gliomas. As CEBPA is located on chromosome 19q13.11, the deletion of chromosome 19q likely inhibits Galectin-9 transcription by downregulating CEBPA expression. Due to the absence of ligands, the immunosuppressive effect of TIM-3 was significantly attenuated. This may explain the better prognosis of glioma patients with 1p/19q codeletion. To further investigate whether the chromosome 1p deletion also participated in the regulation of CEBPA and Galectin-9 expression, we also analyzed single-cell sequencing data from the GSE70630 and GSE89567 databases. t-SNE was performed to evaluate CEBPA and Galectin-9 expression levels in glioma cells with different 1p/19q codeletion statuses. The results revealed significantly reduced CEBPA and Galectin-9 expression levels in most glioma cells with 1p/19q codeletion (**Figure 5C**). The same phenomenon was found in glioma cells with only chromosome 19q deletion (**Figure 5C**). However, glioma cells with intact chromosome 1p/19q showed a significantly increased proportion of cells with high CEBPA and Galectin-9 expression levels (**Figure 5C**).

### CEBPA, Galectin-9, and TIM-3 Protein Expression Levels by IHC

**Figure 6:**
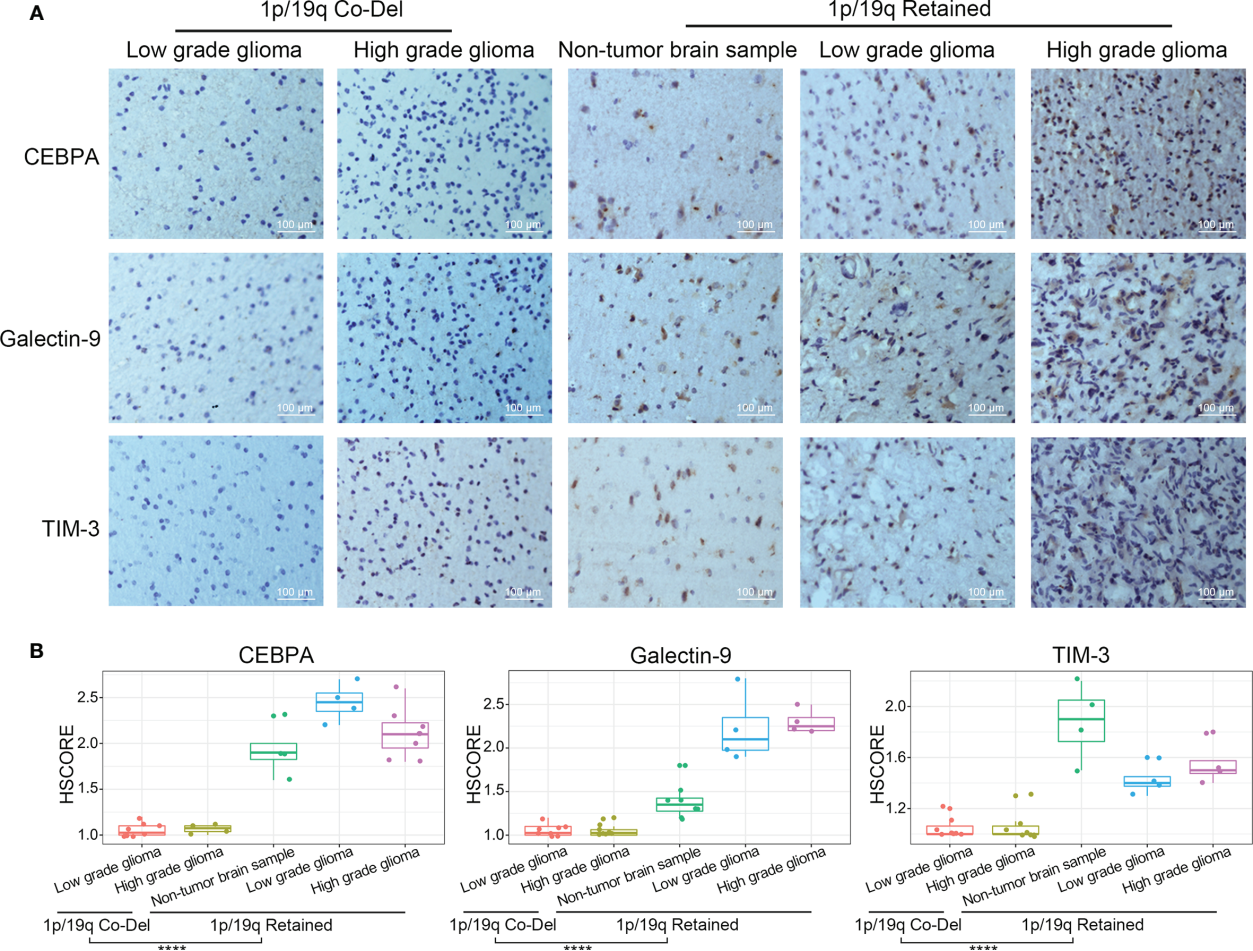
TIM-3, Galectin-9, and CEBPA expression levels evaluated by IHC protein staining in glioma samples stratified by WHO grade and 1p/19q codeletion status. **(A)** IHC staining of CEBPA, Galectin-9 and TIM-3 in glioma samples stratified by WHO grade and 1p/19q codeletion status. **(B)** Differential analysis of HScore of IHC staining of CEBPA, Galectin-9 and TIM-3 in glioma samples. The significance of the difference between the two groups was verified by *Student’s t-test*. ^****^p < 0.0001.

To further verify the results of the bioinformatics analyses described above, IHC protein staining was performed in an independent group of glioma patients from the CGGA database. After stratifying glioma samples according to WHO grade and 1p/19q codeletion status, we found lower expression levels of CEBPA, Galectin-9, and TIM-3 proteins in whole grade glioma samples with 1p/19q codeletion compared with those in samples with retained 1p/19q or normal tissues (**Figures 6A, B**). Collectively, we proved that 1p/19q codeletion status had a significant impact on the tumor immune microenvironment of gliomas at mRNA and protein levels.

### The Decreased Galectin-9 Regulated by CEBPA Can Improve the Efficacy of Cytotherapy

**Figure 7:**
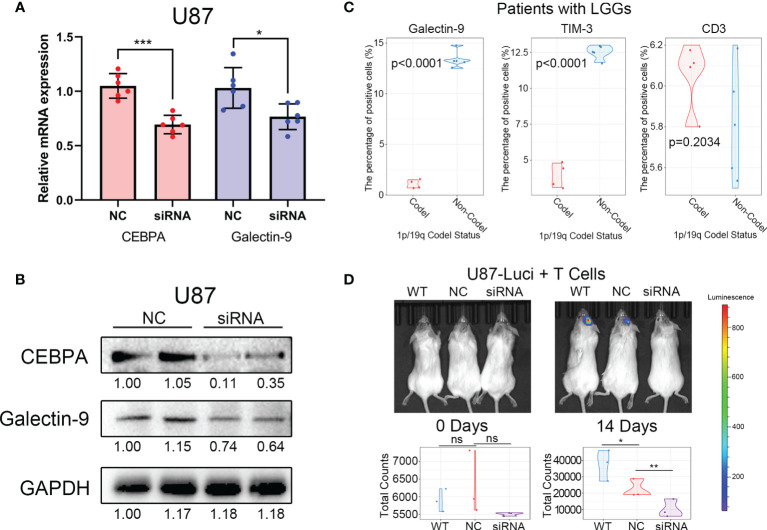
Decreased CEBPA expression leads to a decrease in Galectin-9 expression and an increase in the efficacy of cytotherapy. **(A)** Results of RT-PCR showed that the decrease of CEBPA leads to a significant decrease in the mRNA expression of Galectin-9 in U87. **(B)** Results of western blot showed that the decrease of CEBPA leads to a significant decrease in protein expression of Galectin-9 in U87. **(C)** Results of flow cytometry showed that the proportion of Galectin-9 and TIM-3 positive cells in patients with 1p/19q codel LGGs was significantly lower than that in patients with 1p/19q non-codel LGGs. There was no significant difference in the proportion of CD3 positive cells between the two groups. **(D)** Results of *in vivo* fluorescence imaging showed that the decrease of CEBPA can enhance the killing effect of T cells on tumor cells. The significance of the difference between the two groups was verified by *Student’s t-test*. ^*ns*^ *p > 0.05*, ^*^p < 0.05, **p < 0.01, ^***^p < 0.001.

To further verify the regulatory relationship between CEBPA and Galectin-9, biochemical studies were performed. *In vitro* experiments confirmed that knockdown of CEBPA can significantly decrease in expression of Galectin-9 at the transcriptome level and protein level (**Figures 7A, B**). Furthermore, 4 samples from 1p/19q codel LGG and 5 samples from 1p/19q non-codel LGG were collected for flow cytometry experiments. The proportion of cells expressing CD3, TIM-3 and Galectin-9 were tested by Flow Cytometer. The results showed that the proportion of CD3+ cells was no significant correlated with the 1p/19q codel status of LGG, and the positive ratio of TIM-3 and Galectin-9 were significantly lower in 1p/19q codel LGG (**Figure 7C**).

Cytotherapy, as a new type of tumor immunotherapy, is coming into focus recently (29). Therefore, the difference in efficacy of cytotherapy in 1p/19q codel and non-codel tumors were explored. Luciferase labeled glioma tumor cells with/without CEBPA knock down and T cells were mixed (1:1) and then transplanted into the intracranial of immunodeficient mice. The growth of intracranial tumors in mice was monitored weekly by *in vivo* fluorescence imaging. The results showed that tumors with CEBPA knock down (simulate the 1p/19q codel tumor) can improve the inhibition of T cells on tumor cells (**Figure 7D**).

## Discussion

The 2016 WHO Classification of Tumors of the CNS included several tumor-specific molecular alterations in its classification and diagnosis of gliomas (7). Among them, 1p/19q codeletion status was included in the stratification of oligodendroglioma. Several studies have demonstrated the significant prognostic value of 1p/19q codeletion in WHO subgroups of oligodendrocytoma (10, 12). In recent years, immunotherapy has been widely applied in tumors, but many solid tumors show poor immunotherapy effects due to the special immunosuppressive microenvironment (30). Glioma, as an immunologically “cold tumor”, is considered to be highly resistant to immunotherapy (31). However, our study found that patients with 1p/19q co-deleted glioma may benefit from immunotherapy. Our research provided a theoretical basis for the application of immunotherapy in these glioma patients.

The tumor immune microenvironment, including immune cells and immune-related molecules, plays an essential role in glioma occurrence and development (32). The present study mainly explored the association between 1p/19q codeletion and glioma tumor immune microenvironment. In this study, we found that 1p/19q codeletion status did not significantly affect immune cell infiltration. However, the expression levels of immune checkpoint TIM-3 and its ligand Galectin-9 were tightly associated with 1p/19q codeletion. To further explore the specific molecular mechanism of this phenomenon, we assessed four transcription factors of Galectin-9 (AR, CEBPA, CEBPB, and TFAP2C) and found that CEBPA on chromosome 19q played a leading role in the transcriptional regulation of Galectin-9 in gliomas. Moreover, t-SNE analysis in single-cell RNA sequencing data indicated that the loss of chromosome 19q rather than chromosome 1p caused decreased Galectin-9 expression and abnormal TIM-3 function. CEBPA’s regulation of Galectin-9’s transcription and protein has been verified by experimental and clinical samples. Importantly, the killing effect of T cells on CEBPA-decreased tumor cells was significantly increased *in vivo*. This result suggests that patients with 19q deletion may be insensitive to immune checkpoint inhibitors due to TIM-3 and Galectin-9 dysfunction. In contrast, patients with intact chromosome 19q could benefit from cytotherapy.

In addition to TMZ, limited breakthroughs in recent decades have been reported to improve the outcome in the conventional treatment of gliomas (33–36). To change this situation, many efforts have been made to identify novel molecular markers and therapeutic methods for managing gliomas (5, 6, 8, 37–39). However, limited studies have focused on the effect of 1p/19q codeletion on the biological processes and tumor immune microenvironment of gliomas.

Gliomas with 1p/19q codeletion tend to show characteristics of weaker invasive ability and higher therapy sensitivity (40–42). This is one of the main reasons that oligodendrogliomas characterized by 1p/19q codeletion show a clear boundary on magnetic resonance imaging and better clinical prognosis (43). Recently, Chai et al. reported a significant correlation between 1p/19q codeletion and genes involved in cell proliferation, the extracellular matrix, angiogenesis, and DNA injury response (13). Moreover, the oligodendroglioma cells are deficient in microtube-associated gap junction-mediated tumor cell interactions essential for astrocyte invasion, proliferation, and radioresistance (44). However, these studies failed to provide treatment guidance for patients with 1p/19q codeletion, especially immunotherapy. The tumor immune microenvironmental status in many malignant tumors significantly impacts immunotherapy sensitivity (45–47). Our previous studies showed that immune checkpoints such as PD-LI and TIM-3 could suppress T cell function and macrophage-related immune response in gliomas and lead to worse patient prognosis (18, 20). Thus, the negative correlation between TIM-3 and 1p/19q codeletion indicated that gliomas with the loss of chromosome 1p or/and 19q may benefit more from immunotherapy due to the altered immune microenvironment. Our study initially confirmed the above viewpoint and could be applied to guide the immunotherapy of patients with glioma.

This study has several limitations. First, although only patients with IDH mutation and WHO II glioma were included in this study, the bias caused by other genetic differences in the results still cannot be eliminated. Second, the directionality of most biological functions cannot be reflected by the corresponding enrichment scores. Therefore, the difference analysis of enrichment scores in different 1p/19q status in this study only indicated the strength of the correlation. The directionality of the correlation still needs further experimental verification. Third, since 1p/19q co-deleted glioma cell lines cannot be established *in vitro*, the results of *in vivo* and *in vitro* experiments in this study need to be further verified in transgenic mice or patients.

In conclusion, to the best of our knowledge, this was the first study to investigate the association between 1p/19q codeletion and glioma tumor immune microenvironment. Comprehensive analysis of RNA sequencing data from the CGGA and TCGA databases and single-cell sequencing data from the GSE70630 and GSE89567 databases showed that the loss of chromosome 19q altered the tumor immune microenvironment by downregulating the immunosuppressive function of TIM-3 and its ligand Galectin-9 in glioma patients with 1p/19q codeletion, which may further affect the tumor sensitivity to chemotherapy and immunotherapy. These conclusions provided new insight into the biological functions of 1p/19q codeletion in glioma and might be an important reference for individualized immunotherapy in glioma patients.

## Data Availability Statement

The original contributions presented in the study are included in the article/**Supplementary Material**. Further inquiries can be directed to the corresponding authors.

## Ethics Statement

The studies involving human participants were reviewed and approved by Beijing Tiantan Hospital Institutional Review Board (IRB). The patients/participants provided their written informed consent to participate in this study. The animal study was reviewed and approved by Beijing Neurosurgical Institute Review Board (IRB).

## Author Contributions

WZ, HW, and WY: conception, supervision, and design of this article. GL, RH, WF, and DW: data analysis and editing the manuscript. FW, MY, and YZ: data collection and organization of Single-Cell RNA-Sequencing data. YC, CP, and TJ: *in vitro* and *in vivo* experiment. All authors contributed to the article and approved the submitted version.

## Funding

This work was supported by grants from National Natural Science Foundation of China (No. 82072768, 81802994), Construction of the Genomics Platform for Chinese People’s Brain Diseases (No. PXM2019_026280_000002), Sino-German Center Cooperation and Exchanges Program (M-0020) and The Medical and Health Technology Innovation Project of the Chinese Academy of Medical Sciences (2020-I2M-C&T-A-024).

## Conflict of Interest

The authors declare that the research was conducted in the absence of any commercial or financial relationships that could be construed as a potential conflict of interest.

## Publisher’s Note

All claims expressed in this article are solely those of the authors and do not necessarily represent those of their affiliated organizations, or those of the publisher, the editors and the reviewers. Any product that may be evaluated in this article, or claim that may be made by its manufacturer, is not guaranteed or endorsed by the publisher.
