# Supplementary material for: Galectin-9/TIM-3 as a Key Regulator of Immune Response in Gliomas With Chromosome 1p/19q Codeletion
Source: Front Immunol. 2021 Dec 8;12:800928. doi: 10.3389/fimmu.2021.800928 (PMC8692744; doi:10.3389/fimmu.2021.800928)
Supplement: Supplementary file 1 [file DataSheet_1.pdf]

Supplementary Material

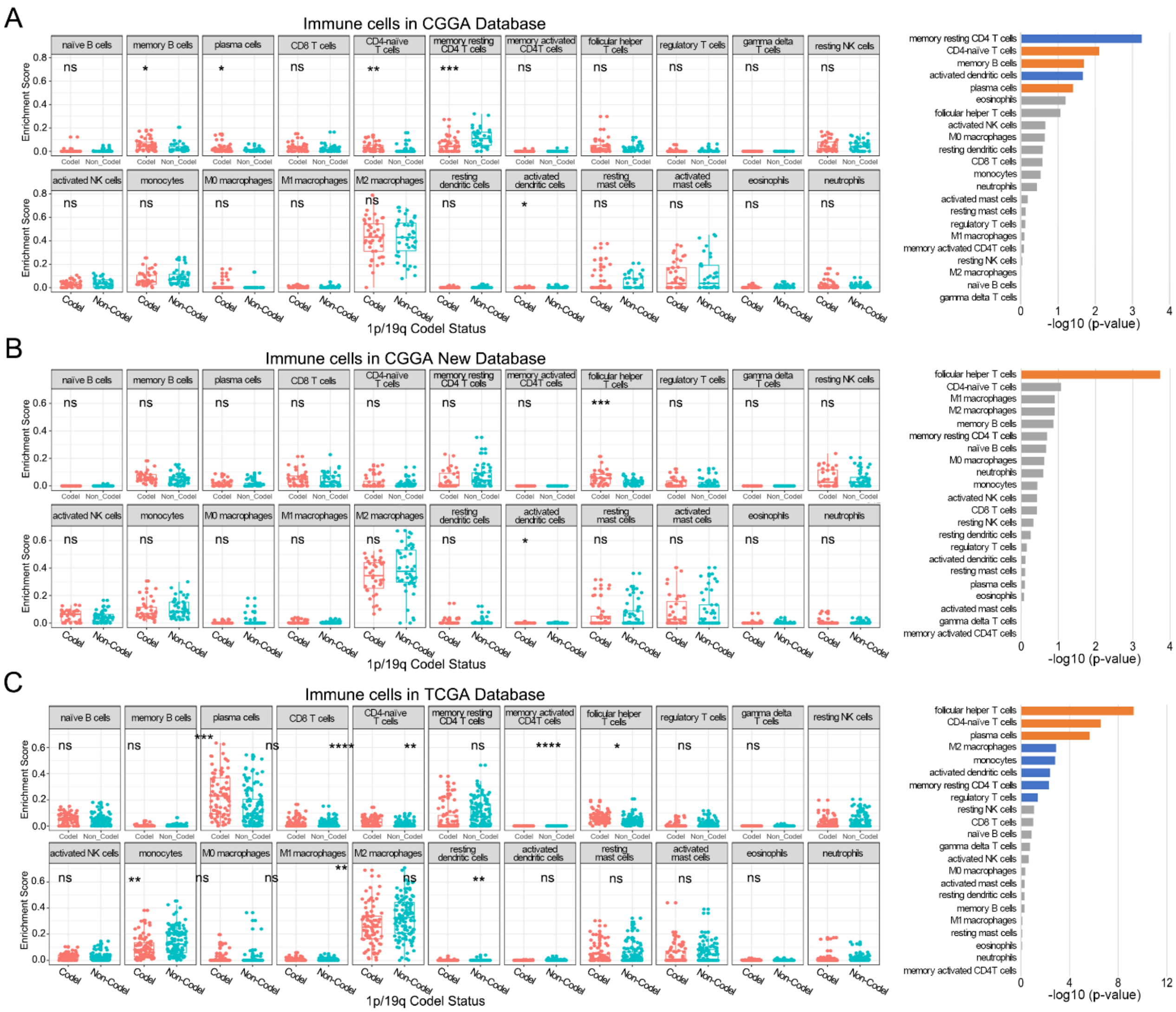

Supplementary Figure 1. The abundance of various types of immune cells evaluated by CIBERSORT in CGGA (A), CGGA NEW (B), and TCGA (C) databases. The significance of the difference between the two groups was verified by *Student's t-test*.

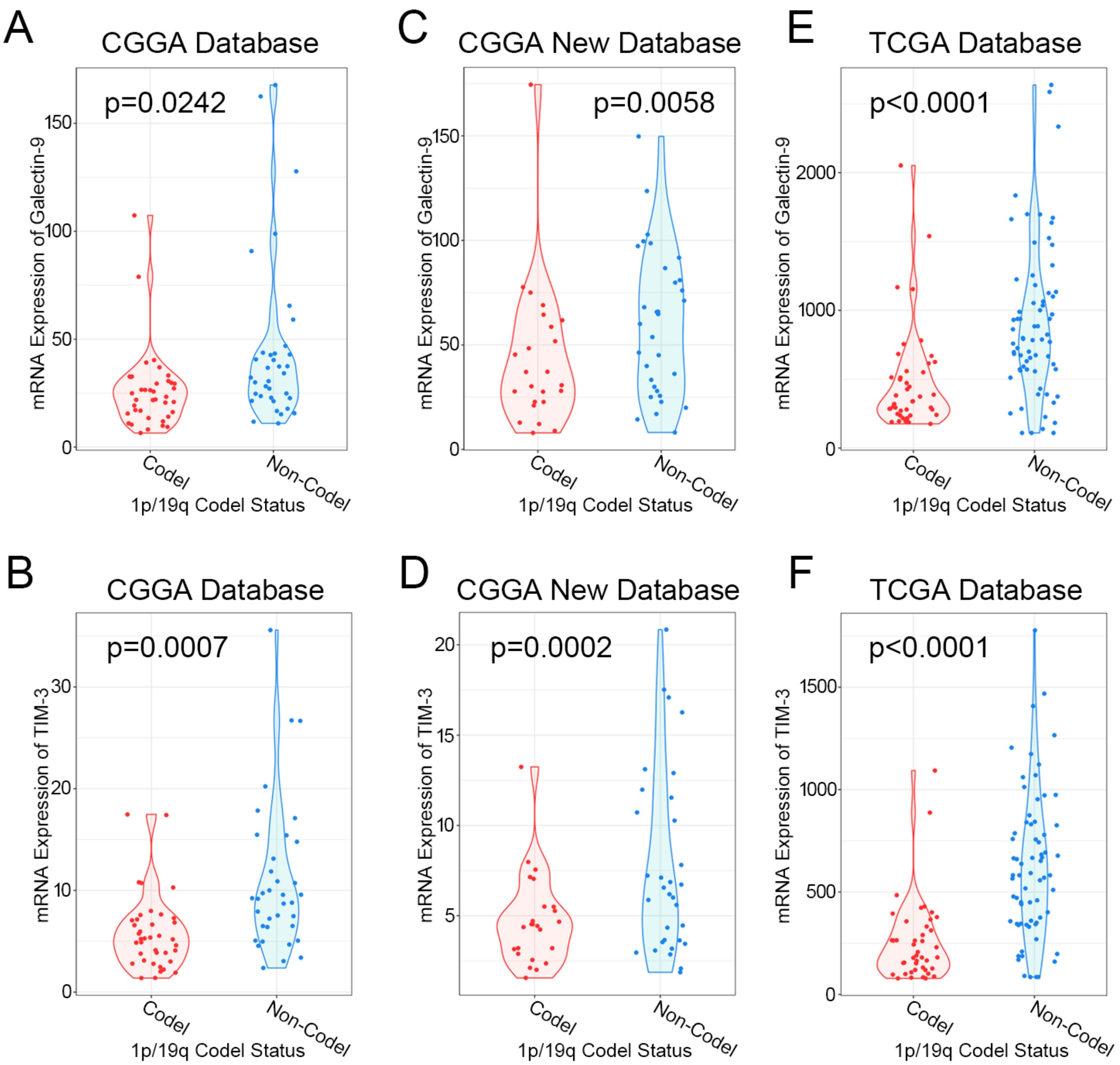

Supplementary Figure 2. Differences in Galectin-9 and TIM-3 expression in glioma patients with different 1p/19q codeletion status in the CGGA (A and B), CGGA NEW (C and D), and TCGA databases (E and F) databases. The significance of the difference between the two groups was verified by *Student's t-test*.

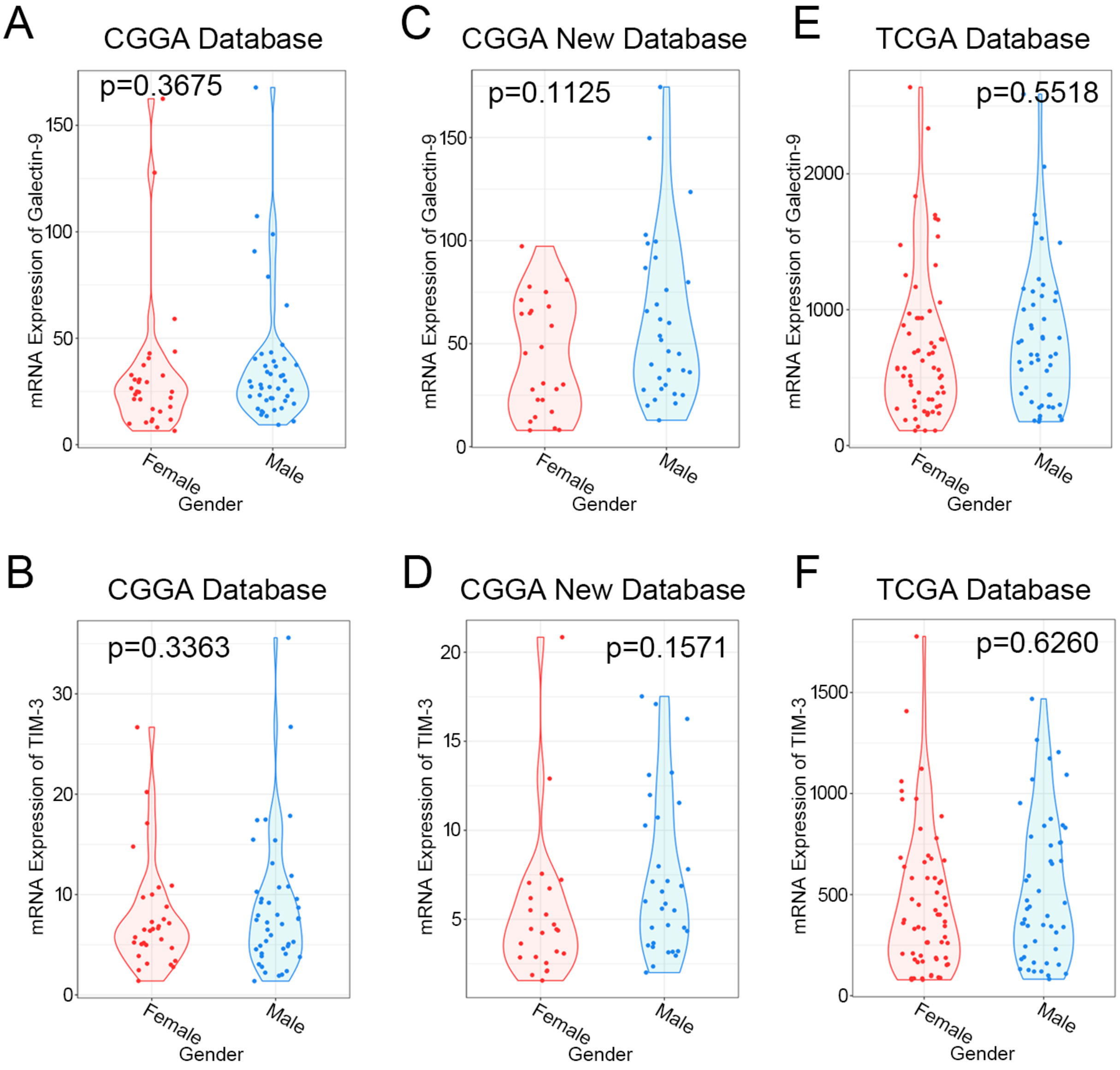

Supplementary Figure 3. Differences in Galectin-9 and TIM-3 expression in female and male glioma patients in the CGGA (A and B), CGGA NEW (C and D), and TCGA databases (E and F) databases. The significance of the difference between the two groups was verified by *Student's t-test*.

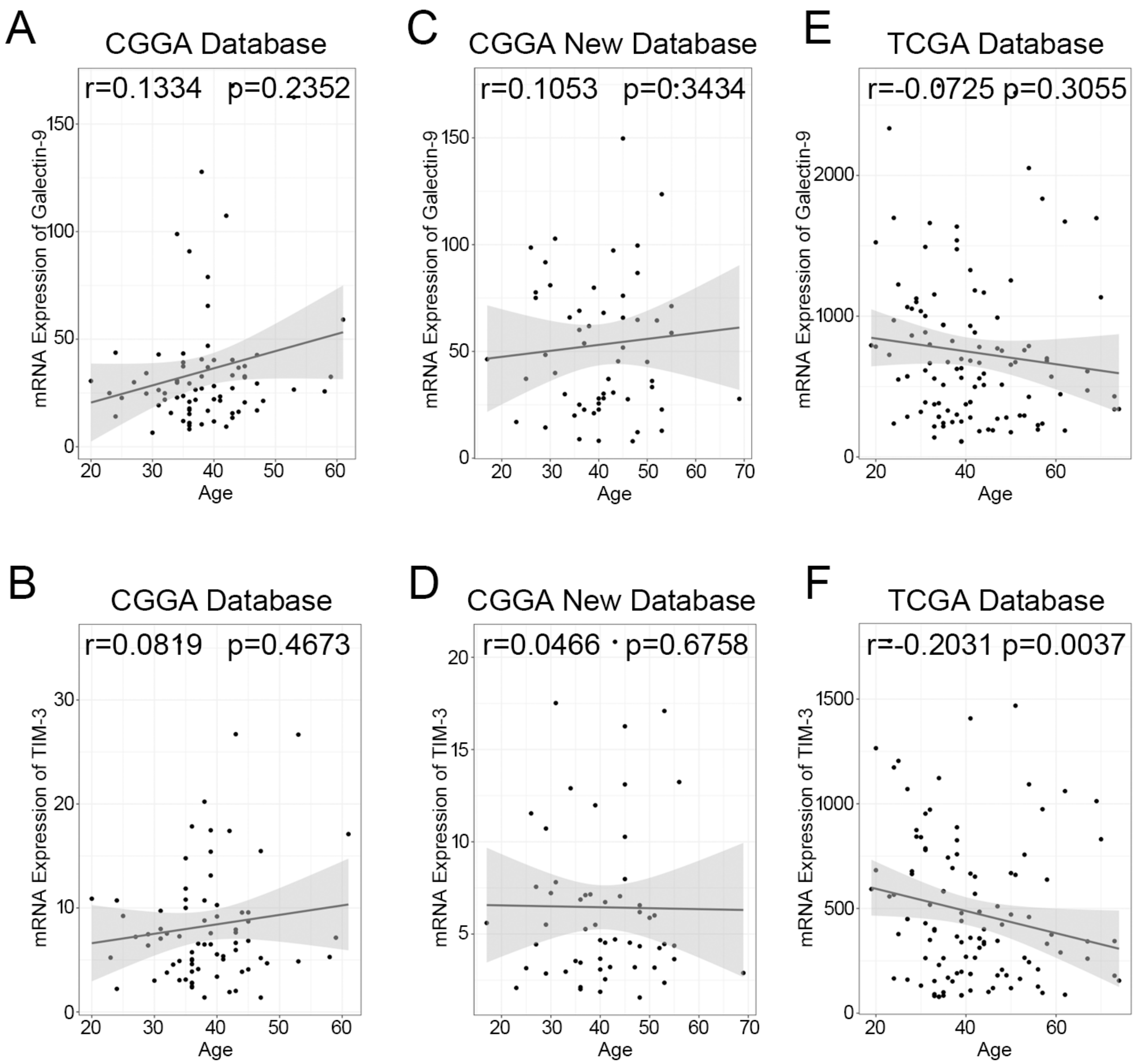

Supplementary Figure 4. Correlation analysis between Galectin-9 and TIM-3 expression and age in glioma patients in the CGGA (A and B), CGGA NEW (C and D), and TCGA databases (E and F) databases. The correlation between Galectin-9 and TIM-3 expression and age was verified by *pearon* correlation analysis.

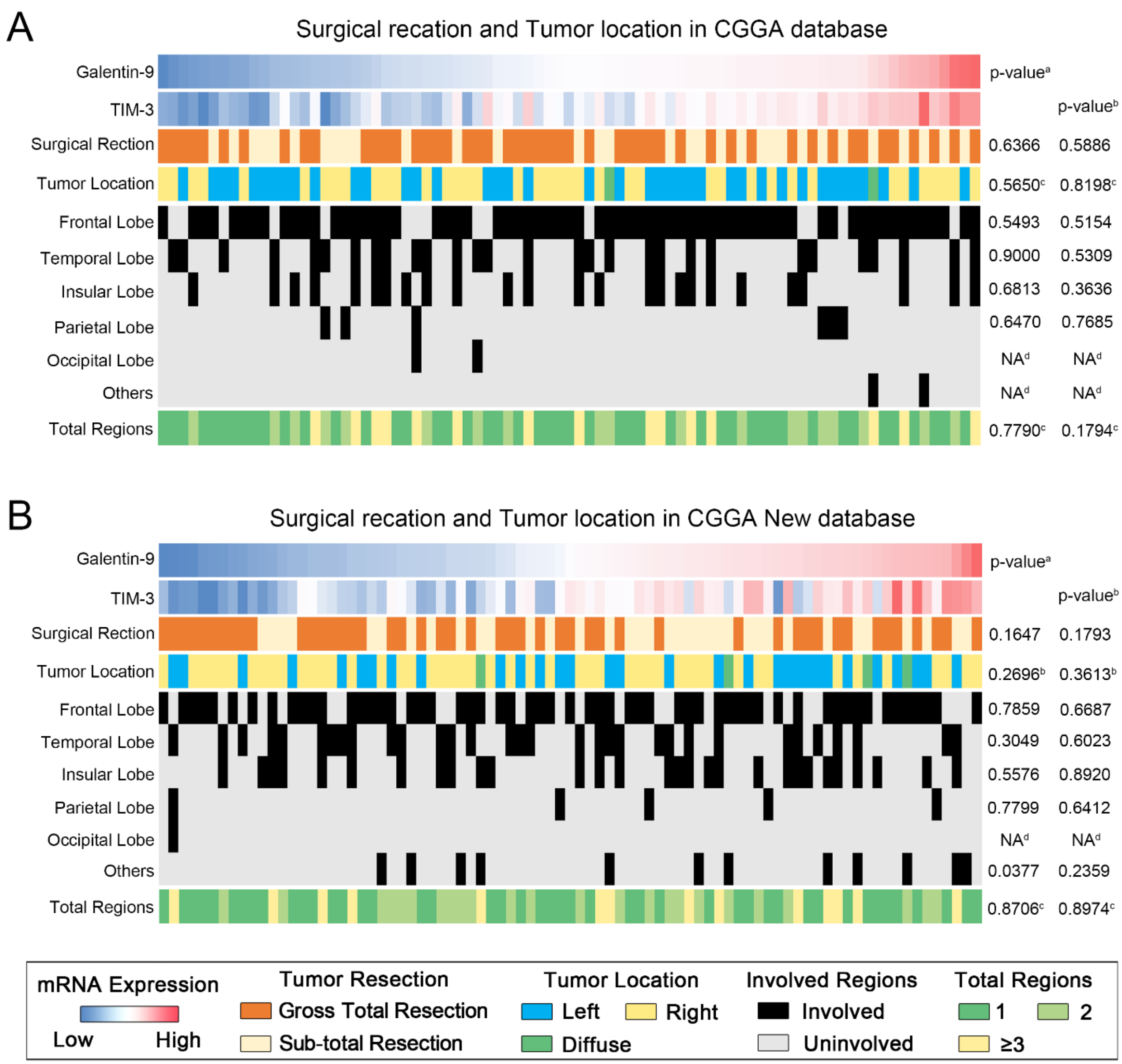

Supplementary Figure 5. Heatmap showing the relationship between Galectin-9 and TIM-3 expression and and tumor involvement and degree of surgical resection in glioma patients in the CGGA and CGGA NEW databases. [a]The significance of the difference expression of Galectin-9 between the two groups was verified by *Student's t-test*. [b]The significance of the difference expression of TIM-3 between the two groups was verified by *Student's t-test*. [c]The significance of the difference expression of Galectin-9 or TIM-3 between the three groups was verified by *One-Way ANOVA*. [d] The difference cannot be statistically tested due to the small sample size.

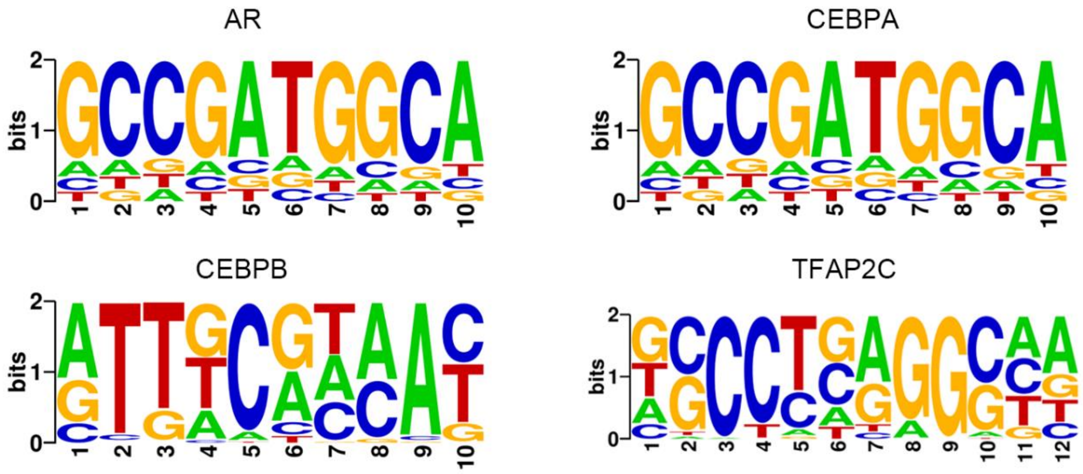

Supplementary Figure 6. The predicted binding sites of four transcription factors (AR, CEBPA, CEBPB, and TFAP2C) on Galectin-9 promotor region.
